# Application progress of artificial intelligence and augmented reality in orthopaedic arthroscopy surgery

**DOI:** 10.1186/s13018-023-04280-9

**Published:** 2023-10-14

**Authors:** Haojie Chen

**Affiliations:** 1 Department of Orthopaedics, The First People’s Hospital of Xiaoshan District, No. 199, Shixin South Road, Chengxiang Street, Xiaoshan District, Hangzhou, China; 2 Xiaoshan Affiliated Hospital of Wenzhou Medical University, Hangzhou, Zhejiang China

**Keywords:** Artificial intelligence (AI), Augmented reality (AR), Arthroscopy surgery, Precise localization

## Abstract

In today's rapidly developing technological era, the technological revolution triggered by the rapid iteration of artificial intelligence and augmented reality has provided brand-new digital intelligent empowerment for orthopaedic clinical operation. Although traditional arthroscopy has been widely promoted globally due to its advantages such as minimally invasive, safety and early functional exercise, it still has deficiencies in precision and personalization. The assistance of artificial intelligence and augmented reality enables precise positioning and navigation in arthroscopic surgery, as well as personalized operations based on patient conditions, which lifts the objective limitations of traditional sports medicine surgery. The integration of artificial intelligence and augmented reality with orthopaedic arthroscopy surgery is still in infancy, even though there are still some insufficient to be solved, but its prospect is bright.

## Introduction

With the leapfrog development of computer technology, artificial intelligence (AI) and augmented reality (AR) technologies have ceaselessly evolved and have been widely applied in various fields such as industry, education and health care. In recent years, as a pioneer in the medical industry exploring the application of high-tech, orthopaedics has taken many “first steps” in the blue ocean of precision medicine with the help of AI and AR technologies. The domestically developed Tianji® orthopaedic surgical AI robot [1] has become the world's only orthopaedic robot system capable of performing limb, pelvic and spinal segment surgeries, with an accuracy leading globally of up to 0.8 mm [2–4].Tianji® robot has been routinely applied in more than 150 medical institutions in China, with a surgical volume exceeding 30,000 cases so far. The world's first AI-driven spine surgery augmented reality (AR) navigation system, HOLO Portal™, already obtained FDA (Food and Drug Administration) approval for surgical guidance with 510(k) clearance in January 2022. However, compared with the mature application of “AI + AR” technology in other fields of orthopaedics, its application in arthroscopic surgery is still in the early stages.

Arthroscopic surgery, which allows surgeons to extend their "eyes" and "hands" into the joint, has been widely performed on joints throughout the body, from large joints such as the hip and knee to small joints such as the interphalangeal joint, owing to its advantages of minimally invasive, safety and early functional exercise since the early twentieth century. Meanwhile, it has limitations such as limited operating space, limited field of view and insufficient surgical precision as is known to all [5]. In recent years, orthopaedic surgeons and engineers at home and abroad have attempted to use AI and AR to promote arthroscopic surgery and have made significant progress. For example, it has improved surgical safety, increased the precision of navigation and operation under the arthroscope, reduced the incidence of surgical complications, reduced intraoperative radiation damage and shortened the duration of surgery, opening up a new situation for the interdisciplinary development of biomedicine and engineer in sports medicine area [6]. This article will provide a comprehensive review of the principles of four parts including AI and AR technology in surgery, the composition of AI and AR surgical systems, the current application of AI and AR in orthopaedic arthroscopy surgery, and the disadvantages and challenges of applying AI and AR in the field of orthopaedic arthroscopy.

## Principles of AI and AR technology in surgery

Artificial intelligence is abbreviated as AI, and its principle can be roughly summarized as combining a large amount of data, mighty computing power and intelligent algorithms to build models that solve specific problems, enabling programs to automatically learn potential patterns or features from the data, thus achieving a thinking process similar to humans [7]. Augmented reality (AR) utilizes computer graphics and visualization technology to generate virtual objects that do not exist in the real world and accurately place them in the real world, presenting users with a new environment with richer perception [8]. Currently, AI technology is mainly applied in the planning and navigation stages of orthopaedic surgical robots. By transmitting patients' X-ray, CT or MRI imaging data to computers before surgery, AI can use difference in texture or colour of vascular angiography and CT or MRI sectional scans to construct 2D or 3D model images mapping the surgical space. Based on this, AI can plan the surgical path, simulate the surgical process, analyse and process intraoperative images in real time, autonomously segmenting and labelling anatomical structures, and planning more scientifically reasonable surgical paths based on recognized key points [9, 10]. Different from AI, AR systems mainly use non-realistic rendering or reverse reality technology to render the established virtual model and present it on the display, accurately reintegrating the model image and guidance information into the real scene, dynamically displaying the anatomical relationship between surgical instruments and surgical sites, and accurately assisting surgeons in completing surgical operations, realizing the visualization and deep perception of the primary surgeon's information [11]. The development and integration of AI and AR in surgical devices are in varying degrees, but the combination of AI and AR systems can achieve a synergistic effect described as “1 + 1 > 2”, which namely enabling intelligent matching and precise positioning, three-dimensional dynamic observation, display of the depth and angle of the surgical path, avoiding dangerous areas, providing rich surgical image information and monitoring the surgical environment and process. Currently, it has been applied in orthopaedic surgery, trauma surgery and spinal surgery with excellent results [12].

## Composition of AI and AR surgical systems

AI surgical systems typically consist of medical imaging module, tracking and positioning module, and display module [13]. Firstly, various high-resolution imaging examination data form the basis for high-definition modelling in the imaging module. Secondly, the working mode of the tracking and positioning module can be divided into the following types: magnetic field positioning, ultrasound positioning, optical positioning and mechanical positioning. Optical positioning has the highest precision and is currently the most commonly used positioning method in orthopaedic AI surgery. The tracking and positioning module mainly includes sensors and locators to achieve real-time tracking and positioning of the relative position between surgical instruments and lesions. The sensors are usually pre-fixed on the patient, and surgical tools then can be tracked by the locator; therefore, the locator receives signals from the sensors in real time. After processing the information based on software algorithms, the relevant information could be transmitted to the image display module. Finally, the image display module mainly implements two functions: spatial registration and image fusion. Spatial registration is to unify preoperative imaging, intraoperative imaging, surgical instruments and lesions in one spatial coordinate system by software algorithms. Image fusion is to comprehensively display the registered medical images and the position information of the tracking and positioning module to achieve a more intuitive and clear display effect [14]. There also needs to be equipped with operating arms, eccentric mechanisms, specially made surgical instruments and other hardware facilities if it was an AI robot.

AR surgical systems consist of three core components: virtual image or environment modelling, registration of the virtual environment with real space and display technology combining the virtual environment with the real world [15]. The first two components are essentially the same as the AI surgical system in terms of hardware and software composition. The main difference between AR and AI technology lies in the display technology combining the virtual environment with the real space. The types of AR display technology can be roughly divided into head-mounted display (HMD), enhanced optical system, enhanced external display, enhanced window display and image projection. HMD can overlay the virtual environment on the real world in the user's field of view (optical perspective) or on the video source in the real environment (video perspective). Enhanced display is displaying virtual content on the video of the real world through an independent screen. Optical enhanced display refers to direct enhancement of the eyepiece of the operating microscope. Window enhanced display is to place a semi-transparent screen directly above the surgical site, allowing virtual objects to be displayed straight on the screen above the real object and be directly projected onto the patient through a projector.

## Current status of application of AI and AR in orthopaedic arthroscopic surgery

### Shoulder arthroscopy

Jung et al. [16] compared the differences in anchor placement effects when five operators performed conventional and AI-assisted arthroscopic surgery on prosthetic models and cadaver shoulders. In prosthetic models, the experimental tasks included anchor placement in the rotator cuff footprint and suture knotting operations. A motion analysis camera system was used to track the hand movements of the surgeons, and the surgical performance indicators included total path length, number of operations and duration of surgery. In the cadaver experiment, the feasibility of AI-assisted anchor insertion was verified by comparing the repeatability and reproducibility of anchor angles inserted by three experts. The results shows that there was no significant difference in total path length, number of operations and time between conventional shoulder arthroscopy and AI-assisted shoulder arthroscopy systems in the prosthetic models. However, in the cadaver experiment, the statistical data of the anchor insertion angle show that AI assistance enabled both novice and expert surgeons to repeatedly insert anchors at angle close to the predetermined target, with an angle error < 2° (*P* < 0.05), which indicates that AI assistance can improve the accuracy of anchor insertion, allowing even inexperienced surgeons to easily insert suture anchor along the correct direction, significantly improving the surgical outcomes of beginners and achieving high repeatability and reproducibility of anchor insertion.

Critical shoulder angle (CSA) refers to the angle between the line connecting the upper and lower borders of the glenoid cavity and the line connecting the lower border of the glenoid cavity and the outer border of the acromion. Moor et al. [17–20] believe that CSA describes the relationship between acromion lateralization and tilt of glenoid cavity, which has been proved to be an effective predictor of shoulder joint pathological development. Numerous studies have shown that shoulders with a CSA less than 30° may be associated with osteoarthritis, while shoulders with a CSA greater than 33°–35° are related to rotator cuff tears (RCT). Nevertheless, how to avoid insufficient CSA or excessive reduction in shoulder arthroscopy acromioplasty remains a challenging problem at present. The team led by Yang et al. [21] proposed a computer image-guided precise acromioplasty (CIG-PAP) technique, which is a personalized treatment based on three-dimensional (3D) planning. It utilizes modelling techniques common to AI and AR to premark and measure bone resection on the model, enabling the reduction of larger CSA to the desired range (30°–33°) during surgery. CIG-PAP is particularly suitable for patients with an initial CSA greater than 35° combined with preoperative RCT and can bring clinical benefits to patients in combination with arthroscopic rotator cuff repair.

### Elbow arthroscopy

Guo et al. [22] explored the efficacy of AI-assisted arthroscopy in the treatment of primary elbow osteoarthritis with stiffness. Preoperatively, AI is used to simulate elbow joint motion from 0° extension to 140° flexion to determine the location and extent of osteophyte impingement, and 3D modelling is used to display the amount and degree of osteophyte removal needed in the anterior and posterior directions of the elbow joint. Afterwards, arthroscopic visualization is in order to assess the effect of elbow joint release and osteophyte removal. Visual analog scale (VAS), Mayo elbow performance score (MEPS) and elbow range of motion are for the purpose of evaluating elbow joint function pre- and postoperatively. Statistical analysis of the data at the last follow-up revealed significant improvement in VAS scores, MEPS scores and elbow range of motion compared to preoperative values, indicating significant pain reduction and functional recovery.

Shiode et al. [23] adopted an AI-assisted system in elbow arthroscopy for joint debridement and found that the accuracy of using the AI-assisted system for elbow arthroscopic debridement was the same as that in other joints.

In elbow arthroscopic debridement, the identification and precise removal of impinging bone lesions present technical challenges. Shigi et al. [24] utilized an AI system combined with preoperative three-dimensional evaluation of impinging bones to provide real-time tracking of surgical instruments and impinging lesions. The registration procedure was tested using resin bone models of three patients with elbow osteoarthritis. Digitalization of bone surface points was conducted using a navigation pointer during arthroscopy. The total registration accuracy for the humerus and ulna was 0.96 mm and 0.85 mm, respectively. There was no significant difference in registration accuracy of the humerus and ulna among the three observers during arthroscopy, confirming the feasibility of AI-guided navigation in arthroscopic surgery.

### Wrist arthroscopy

Scaphoid injuries are extremely common in wrist injuries as well as have a high non-union rate. Statistics show a non-union rate of 40% in conservatively treated scaphoid fractures with cast fixation. Due to the small size, concealed location, complex and irregular three-dimensional morphology of the scaphoid, it is difficult to insert screws accurately during traditional internal fixation surgery, which can easily lead to complications such as joint cartilage wear and delayed or non-union fractures [25–29]. The team led by Fang et al. [30, 31] attempted autogenous bone grafting under wrist arthroscopy combined with AI robot-guided placement of compression screws to treat scaphoid non-union and Herbert-type D1 scaphoid fractures. Both the postoperative Mayo function score and VAS pain score were satisfactory, the wrist range of motion and grip strength significantly improved as well.

Professor Liu et al. [32] screened subacute scaphoid fractures with wrist arthroscopy and performed screw placement under AI robot navigation. The postoperative follow-up showed excellent results with satisfactory positioning and length of internal fixation as well as good fracture reduction, proving the technical feasibility of wrist arthroscopy under AI assistance.

Jeung et al. [33] hold the opinion that the difference between intraoperative joint conditions and preoperative CT/MR images since the movement applied during the operation results in inaccurate targeting for surgical approaches. To accurately display hidden wrist bones in arthroscopic images, they proposed a surgical guidance system that utilizes a new bone displacement compensation method employing non-invasive reference markers, which greatly eliminates AR errors caused by wrist traction. Furthermore, this system allows for precise AR display of hidden bones and expands the limited field of view of the arthroscope. The proposed bone displacement compensation method can also be applied to other joints, such as the knee or shoulder, by representing their skeletal movements by corresponding virtual links. Additionally, the motion of the joint skin during surgery can be measured using non-invasive reference markers in the same way as the wrist joint.

### Hip arthroscopy

Cam morphology, which refers to a shape of the cam, is one of the important factors leading to hip impingement. The teams led by Nakamura et al. [34] and Kobayashi et al. [35], respectively, had taken advantage of computer navigation for preoperative planning and intraoperative navigation of hip arthroscopy in the treatment of hip impingement, as allows accurate identification of impingement points and the location and extent of cam lesions, thereby enabling precise femoral head and neck osteoplasty.

Stražar et al. [36] considered that the use of AR system in hip arthroscopy was highly effective with obvious advantages. Prior to surgery, three-dimensional reconstruction of the hip joint was performed capitalizing upon low-dose CT scan. The EBSVR software is applied for pelvic examination with *α* and *γ* angles serving as anatomical parameters for femoral head sphericity. After identifying the impingement area, the improvement of range of motion (ROM) following virtual resection was predicted. The model of the preplanned bone volume resection was then transferred to the GUIDING STAR® VR surgical navigation system based on electromagnetic tracking for the purpose of precise surgical positioning and meticulous operation. This approach reduced surgery time and intraoperative fluoroscopy, while achieving near-perfect femoral head sphericity.

Abe et al. [37] deemed that even though AI-assisted surgery can improve the accuracy of arthroscopic bone and cartilage shaping procedures, there are few clinical studies evaluating their accuracy. Their study focused on patients with cam-type femoroacetabular impingement (FAI) who underwent AI-assisted arthroscopic surgery. Three-dimensional models of the femur were constructed based on CT data for each patient, virtual cam resection models were generated preoperatively, postoperatively femoral models were reconstructed based on CT data, and the above three models for each patient were superimposed manipulating a three-dimensional model registration method. Subsequently comparing the contours of the bone resection areas in each model, it was found that AI-assisted arthroscopic bone and cartilage shaping procedures showed good accuracy. Despite the help of intraoperative navigation, incomplete resection in the anterior superior portion of the femur was more common than over-resection.

### Knee arthroscopy

Arthroscopic reconstruction of the cruciate ligament under guidance is one of the earliest arthroscopic procedures that have been assisted by AI technology, as is mainly used for the selection of reconstruction sites and bone tunnel directions. In 2006, Hong et al. [38] used a computer navigation system based on X-ray imaging to assist arthroscopic anterior cruciate ligament (ACL) reconstruction. They held that AI-assisted navigation provided data which were closer to the anatomical reconstruction position, allowing for more accurate placement of the femoral and tibial tunnels; furthermore, the technique was considered safe and feasible. Wang and Peng [39] made use of AI to assist arthroscopic ACL reconstruction surgery, in which they utilized bone tunnel navigation based on anatomical landmarks and kinematic data of the knee joint. The study concluded that this method provided accurate positioning and excellent postoperative outcomes. Zhang Kai's team [40] performed reconstruction of injured with a vascular pedicle patellar ligament. They compared the consequences of ACL reconstruction between AI-assisted arthroscopy and traditional arthroscopy by measuring the bone tunnels in postoperative CT scans. The results suggested that the AI group had higher positions of both the femoral and tibial tunnels than the traditional group, and the AI group had significantly higher Lysholm scores at 3, 6 and 12 months postoperatively, fewer fluoroscopy procedures during surgery and tighter fit with the distance less than 2 mm between the posterior wall of the tunnel and the proximal posterior cortex of the tibia while mild rupture of the exit site of the posterior wall in the traditional group. These results suggested that AI-assisted arthroscopic ACL reconstruction is more accurate, safe, anatomical and ideal in terms of outcomes compared to traditional arthroscopy. Qiu et al. [41] found no statistical difference in knee joint stability and function between AI-assisted and manual ACL reconstruction. However, AI-assisted ACL reconstruction resulted in femoral tunnel positioning that was closer to the anatomical position, while tibial tunnel positioning did not show significant differences compared to manual positioning. Hu et al. [42] leveraged 30 fresh frozen adult knee joint specimens to build three-dimensional models based on CT data and performed tibial tunnel reconstruction under the real-time monitoring of an electromagnetic navigation system according to the 3D models. They measured the sagittal angle, tunnel length and exit position of the tibial tunnel and then draw a conclusion that the planned tibial tunnel angle and length matched the measured results with high accuracy. In addition, the system was found to be convenient and effective as an assisting positioning method. Zhang et al. [43] conducted a retrospective cohort study comparing the clinical efficacy of AI-assisted arthroscopic ACL reconstruction with that of traditional arthroscopy. They found that the AI-assisted group wasted slightly longer time, but was able to prepare bone tunnels with good positioning and direction in one step, achieving similar joint stability and functional recovery as the traditional arthroscopy group. Yang Xiao [44] successfully completed the first case of AI-assisted arthroscopic reduction and fixation for children's avulsion fracture of posterior cruciate ligament (PCL) arrest in China, greatly reducing intraoperative radiation exposure for the children and minimizing the impact on the normal growth and development of the proximal tibia.

The disadvantage of knee arthroscopy is the lack of depth information and potential obstruction of the field of view. To address these issues, engineer Ma [45] and his team developed an AI navigation system for arthroscopy based on self-localization technology. They fused visual and inertial data to estimate the arthroscope's pose on the basis of visual inertial stereo odometry and used virtual visualization to provide flight views and global localization views for surgical guidance as the same time. The flight view provided surgeons with a method to navigate the arthroscope within internal anatomical structures in a manner of virtual camera perspective. The global localization view displayed the arthroscope's pose relative to the preoperative model in a transparent manner. His team also developed a flexible calibration method to transform the real pose of the arthroscope into a virtual visual rendering framework for arthroscopic navigation systems with self-localization information. This system expanded the working range, improved the robustness of rotational operations and meanwhile had great potential for medical applications by eliminating the need for external tracking devices or added markers.

Raposo et al. [46] have made a technical upgrade to the current registration system. They manipulated visually recognizable markers attached to both the skeleton and arthroscopic tools to estimate their relative poses. Then they aligned the preoperative anatomical images of the patients with a set of reconstructed contours of the bone surface obtained through instrument contact using a state-of-the-art registration algorithm. Experimental validation based on ex vivo data indicated that the method achieved precise registration of the preoperative model with the skeleton, as had the advantages of high accuracy and short time consumption and did not require additional incisions or equipment, making it an attractive alternative AR solution.

### Ankle arthroscopy

The team led by Xu et al. [47] retrospectively analysed the difference in the effect of reduction and internal fixation for neck fracture of talus under AI robot-assisted arthroscopy and traditional arthroscopy. The outcomes demonstrated that the AI group had better postoperative ankle joint function assessed by AOFAS score and lower VAS pain score compared to the control group, which were satisfactory and worth promoting. Cao et al. [48] applied the Tianji® orthopaedic robot AI navigation system combined with ankle arthroscopy technique for internal fixation of Hawkins II type neck fracture of talus. There were no complications such as incision infection or avascular necrosis of the talus, and postoperative imaging confirmed satisfactory internal fixation and fracture reduction. The average AOFAS score at the latest follow-up was up to 91.0. The author considered that the method has the advantages of minimally invasive, precise reduction and fixation, fewer postoperative complications together with positive short-term efficacy.

During ankle arthrodesis under arthroscopic surgery, even experienced surgeons often spend a certain amount of time and multiple attempts to reach the predetermined target position while performing Kirschner wire drilling. To address this issue, Duan et al. [49] imported ankle joint DICOM data obtained through CT examination into MIMICS software and utilized 3D printing to design personalized guides afterwards. The control group used Kirschner wire drilling based on the surgeon's previous experience, while the experimental group used the guide to drill two 2 mm Kirschner wires with the position of the wires confirmed by taking a C-arm X-ray before inserting hollow screws. As a result, the application of 3D printing personalized guides assisted accurate drilling in ankle arthrodesis, saving approximately 2 min of surgery time and reducing intraoperative radiation. There were no significant complications in either group during or after surgery. Postoperative X-ray confirmed bone fusion in all cases, confirming that this technique does not affect surgical outcomes.

## Limitations and challenges of AI and AR in orthopaedic arthroscopy

In spite of significant advantages of AI and AR technologies over traditional arthroscopic surgery, there are still some unresolved issues range over: 1. there is an insufficient interdisciplinary collaboration between biomedicine and engineer in the development of AI and AR hardware and software, without fully utilizing the clinical expertise of specialists. 2. AI and AR surgical equipment development is still in the initial stage, whose accuracy, safety, portability and reliability need to be further improved. 3. System updates, algorithm upgrades and firmware updates regularly require network connectivity, posing risks of patient information leakage. It is necessary to consider establishing legal and ethical regulations for AI medical data [50]. 4. The navigation reference frame and the patient's surgical area must be firmly fixed, as any movement or misalignment during registration probably occur [51]. 5. Intraoperative manipulations often cause deformation or displacement of soft tissues, making it difficult to fully reproduce preoperative planning [52]. 6. The defining of truth labels can be significantly influenced by subjective factors in deep learning of navigation algorithms and then bring about result biases, highlighting the need for more objective standards such as intraoperative verification and pathological examination [53]. 7. Current AI surgical robots are only playing the role of assistant of surgeons. Moreover, decision-making power remains in the hands of the surgeon, the optimal balance between AI intelligence and surgical safety. 8. The information registration, data acquisition as well as image registration processes of navigation tools are complex and can delay the duration of the surgery by 5–7 min [54]. 9. Most AI-assisted arthroscopic surgeries require additional incisions for fixing reference frames, increasing patient trauma [55]. 10. Non-rigid transformations got regularly neglect in image fusion. 11. Learning navigation techniques requires rigorous training with a long learning curve and difficulty of operation. 12. Even if AI and AR technologies combining with arthroscopy systems developed for small joints such as the finger joint is recognized to be able to achieve more precise arthroscopic surgery, as still faces high technical barriers, limited supporting equipment and a lack of sufficient data. 13. The procurement and operational costs of AI and AR devices are high, potentially increasing the surgical expenses for patients. 14. Effective frameworks need to be established for the standardized collection and secure management of multi-source, multi-modal, homogeneous and heterogeneous medical data. It is equally important to design new algorithms for small or limited datasets that can learn independently [3]. 15. The lack of open-source basic algorithms and communication protocols makes standardization and widespread adoption difficult [56].

## Summary and prospects of AI and AR applications in orthopaedic arthroscopy

In recent years, AI and AR technologies have developed rapidly and have given rise to related surgical assistive devices, greatly promoting the advancement of orthopaedic arthroscopy. The application of AI- and AR-assisted technologies in orthopaedic arthroscopy allows for personalized surgical planning based on individual patient characteristics. This demonstrates the advantages of intelligent empowerment technology, personally summarized as the “3 FOURs” means “four increases, four reductions and four transformations”. The “four increases” contains more accurate intraoperative positioning, precise operation, closer approximation to anatomical reconstruction and reduction and improved surgical safety. The "four reductions" involves reduced surgical risks, shortened operative time, decreased intraoperative radiation and lowered risk of postoperative complications. The "four transformations" consists of visualization of blind spots, minimization of invasive conventional surgery, securitization of complex procedures and intelligentization of key operations.

The transformational application of AI and AR in the healthcare industry has been identified as a key area for future development by numerous countries and regions. According to the latest annual report released by reputed research firm—ReportLinker, the global healthcare AI market is projected to grow from $14.6 billion in 2023 to $102.7 billion in 2028, with a compound annual growth rate of 47.6% during this period. With the rapid development of related technologies and the establishment of industry standards, it is believed that AI- and AR-assisted orthopaedic arthroscopy systems will gradually overcome the current limitations of insufficient intelligence, algorithmic bias, high technical barriers and expensive equipment. AI- and AR-assisted orthopaedic arthroscopic surgery is expected to achieve more precise and scientific personalized treatment in future operating rooms. Besides the increase in the autonomous decision-making capability of AI, precision, safety and reliability will simultaneously improve. AR device development will be more focused on technology that integrates closely with human organs, such as retina displays and human–machine symbiosis, hence which the performance and portable wearable devices will become research hot spots in the near future. With the combined incubation of global industry demand, financial support and policy driving, the field of AI- and AR-assisted intelligent arthroscopic surgery technology possesses immense development potential and the future looks promising.
